# NMR studies of anion-induced conformational changes in diindolylureas and diindolylthioureas

**DOI:** 10.3762/bjoc.7.140

**Published:** 2011-09-02

**Authors:** Damjan Makuc, Jennifer R Hiscock, Mark E Light, Philip A Gale, Janez Plavec

**Affiliations:** 1 Slovenian NMR Centre, National Institute of Chemistry, Hajdrihova 19, SI-1000 Ljubljana, Slovenia; 2 EN→FIST Centre of Excellence, Dunajska 156, SI-1000 Ljubljana, Slovenia; 3 Chemistry, University of Southampton, Southampton SO17 1BJ, United Kingdom; 4 Faculty of Chemistry and Chemical Technology, University of Ljubljana, SI-1000 Ljubljana, Slovenia

**Keywords:** anion recognition, conformation analysis, host–guest systems, NMR spectroscopy

## Abstract

The conformational properties of 1,3-diindolylureas and thioureas were studied by a combination of heteronuclear NMR spectroscopy and quantum mechanics calculations. NOE experiments showed that the *anti–anti* conformer along the C7–N7α bonds was predominant in DMSO-*d*_6_ solution in the absence of anions. Anion-induced changes in the ^1^H and ^15^N chemical shifts confirm the weak binding of chloride anions with negligible conformational changes. Strong deshielding of ureido protons and moderate deshielding of indole NH was observed upon the addition of acetate, benzoate, bicarbonate and dihydrogen phosphate, which indicated that the predominant hydrogen bond interactions occurred at the urea donor groups. Binding of oxo-anions caused conformational changes along the C7–N7α bonds and the *syn*–*syn* conformer was preferred for anion–receptor complexes. The conformational changes upon anion binding are in good agreement with energetic preferences established by ab initio calculations.

## Introduction

In the last two decades, remarkable efforts have been made in the field of the development of synthetic anion receptors, motivated by prospective applications in recognition, separation, guest inclusion and catalysis [1–13]. The fundamental role of anions in biological and chemical processes drives much of this research. Biomolecules such as the sulfate binding protein [14] and phosphate binding protein [15] employ hydrogen bonds as the key driving force to bind or transport anions through cell membranes. Hydrogen bonding interactions are extensively employed in synthetic anion receptors comprising groups such as amides, pyrroles, indoles, ureas and triazoles, as well as in ammonium, guanidinium and imidazolium moieties used as hydrogen bond donors [16–23]. Amongst neutral anion receptor systems, indole and related heterocycles, such as carbazole, 2,2'-biindole and indolo[1,2-*b*]carbazoles, have recently attracted significant attention [24–31]. Indole contains a single hydrogen bond donor group, which is employed in biological systems to bind anions such as chloride [32] and sulfate [14].

**Figure 1:**
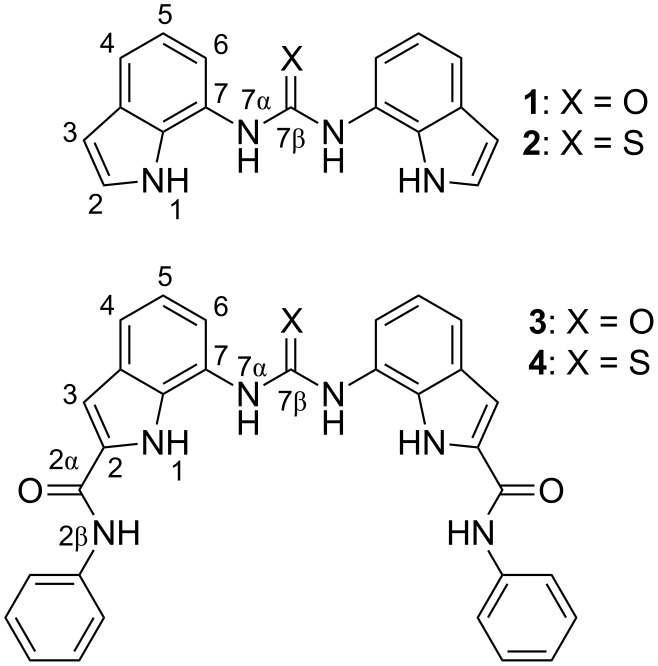
Anion receptors **1**–**4** together with their atomic numbering scheme.

We have recently analyzed the conformational preferences of several 2,7-disubstituted indoles with amide substituents at C2 and urea substituents at C7, which showed preference for distinct conformations in the presence and in the absence of anions [33–35]. In addition, indole and urea groups were strongly involved in hydrogen-bonding interactions with the bound anionic guest, whilst the amide group interacted only weakly with the bound anion. These observations led to the design of diindolylureas and diindolylthioureas [36–38]. These compounds have remarkably high affinities for oxo-anions such as phosphate and sulfate for neutral receptors in DMSO-*d*_6_/0.5% water and have been shown to perturb the p*K*_a_ of bound guests (Table 1) [38–39]. X-ray crystal structures of a variety of complexes with anions revealed the adoption of the *syn–syn* conformation in the solid state upon anion complexation. With the urea analogues, such as **1**, this is accompanied by higher order complex formation with oxo-anions in the solid state. For example, with dihydrogen phosphate, three equivalents of receptor complex to a single anion, which has doubly deprotonated, resulting in the formation of a complex in which twelve hydrogen bonds stabilize the PO_4_^3−^ anion. In solution, the thiourea analogues such as compound **2** show significantly lower affinities for oxo-anions than do the urea analogues. We had previously proposed that this may be due to the larger size of the sulfur atom resulting in a lower propensity of these systems to adopt a planar conformation. Whilst the conformational properties of these compounds have been explored by single crystal X-ray diffraction in the solid-state, a detailed analysis of the conformational properties of the these compounds in solution, in the absence and presence of oxo-anions, has yet to be performed and may help shed light on the high affinity of these systems for oxo-anions. Therefore, in the current work the conformational preorganization of bis-indole receptors **1**–**4** (Figure 1), as well as the conformational changes of these systems upon binding of chloride and several oxo-anions, were studied by NMR spectroscopy and supported by energetic preferences established from ab initio calculations.

**Table 1:** Stability constants of compound **1** measured in DMSO-*d*_6_/0.5% water, DMSO-*d*_6_/10% water and DMSO-*d*_6_/25% water and compound **2** in DMSO-*d*_6_/0.5% water at 298 K by ^1^H NMR titration techniques [37].

Anion^a^	Compound **1** in DMSO-*d*_6_/0.5% water	Compound **1** in DMSO-*d*_6_/10% water	Compound **1** in DMSO-*d*_6_/25% water	Compound **2** in DMSO-*d*_6_/0.5% water

Cl^−^	128	17	–	74
CH_3_CO_2_^−^	>10^4^	774	20	1620
C_6_H_5_CO_2_^−^	>10^4^	521	precipitate	477
H_2_PO_4_^−^	>10^4^	5170	160	1630

^a^Anions added as tetrabutylammonium salts.

## Results and Discussion

### Synthesis

Compounds **1**–**3** were synthesized following a previously reported methodology [36–39]. Compound **4** was prepared by reaction of 7-amino-*N*-phenyl-1*H*-indole-2-carboxamide (0.27 g, 1.07 mM) with 7-isothiocyanato-*N*-phenyl-1*H*-indole-2-carboxamide (0.31 g, 1.07 mM) in pyridine in 27% yield (see Supporting Information File 1 for details).

#### Structural features and NMR chemical shifts

The conformational properties of diindolylureas and diindolylthioureas **1**–**4**, shown in Figure 1, were evaluated by means of NMR spectroscopy. Proton and ^13^C NMR resonances were assigned through 1D and 2D spectra, while ^15^N chemical shifts were determined by ^1^H–^15^N correlations in HSQC and HMBC spectra. Notable ^1^H and ^15^N NMR chemical shifts of **1**–**4** are shown in Table 2. It should be noted that only one set of signals was observed for both indole rings in all four receptors, due to the symmetry of the compounds. The full NMR data set together with ^13^C NMR chemical shifts is reported in Supporting Information File 1.

**Table 2:** Selected ^1^H and ^15^N NMR chemical shifts for **1**–**4** (in ppm).^a^

	H1	H2β	H7α	H2	H3	H6	N1	N2β	N7α

**1**	10.78	–	8.64	7.35	6.44	7.08	136.5	–	102.7
**2**	11.03	–	9.48	7.36	6.46	7.03	136.3	–	124.9
**3**	11.62	10.29	8.97	–	7.49	7.59	134.5	128.6	104.6
**4**	11.68	10.26	9.72	–	7.48	7.39	134.9	129.0	126.6

^a^In DMSO-*d*_6_ at 298 K.

Indole NH proton resonances were found between 10.8 and 11.7 ppm. Thioureido containing compounds **2** and **4** exhibited slight downfield shifts of H1 and H7α with respect to ureido receptors **1** and **3** (Table 2). N1 chemical shifts showed only minor variations as a result of structural differences in **1**–**4**. The most significant differences in chemical shifts between the ureido and thioureido functionalized receptors were observed for H7α and N7α atoms (Δδ_H_ = 0.8 and Δδ_N_ = 22 ppm, Table 2). Compounds **3** and **4** contain phenylamide substituents at C2 and hence two more NH groups (Figure 1). Considerable deshielding of the H3 and H6 resonances was observed in **3** and **4** with respect to the nonsubstituted indole moieties in **1** and **2**. The downfield shift of δ_H3_ was attributed directly to the presence of the phenylamide group at C2. Deshielding of H6 (Δδ 0.4–0.5 ppm) in **3** and **4** with respect to **1** and **2**, respectively, was much larger than the deshielding of H4 (Δδ 0.1 ppm), possibly due to a more efficient conjugation.

#### ^1^H and ^15^N NMR chemical shift changes in **1** upon addition of anions

**Figure 2:**
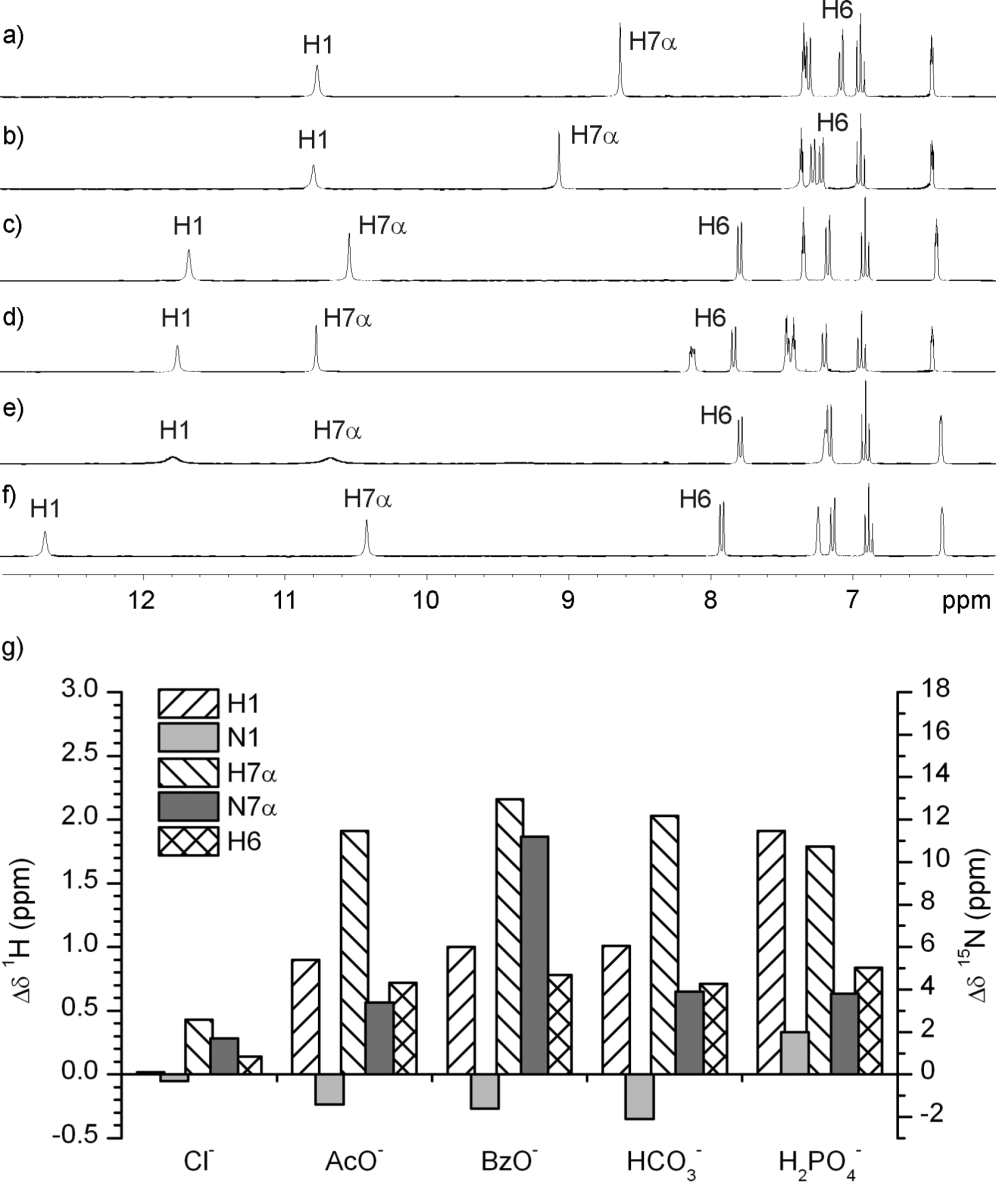
^1^H NMR spectra of **1** in the absence of anions (a) and upon addition of one equivalent of the following anions: Chloride (b), acetate (c), benzoate (d), bicarbonate (e) and dihydrogen phosphate (f). All spectra were recorded in DMSO-*d*_6_ at 298 K. (g) ^1^H and ^15^N NMR chemical shift changes, Δδ = δ (in the presence of anion) – δ (in the absence of anions), induced by addition of one equivalent of different anions to receptor **1**.

Figure 2 shows ^1^H chemical shift changes of **1** in the presence of one equivalent of chloride, acetate, benzoate, bicarbonate and dihydrogen phosphate anions. The protons that are most affected by anion–receptor interaction were found to be H1, H6 and H7α. Only minor Δδ_H7α_ and negligible differences of δ_H1_ and δ_H6_ were observed in the presence of chloride anions (Figure 2a and Figure 2b). The very weak interactions between chloride and **1** could be due to competing interactions of the polar DMSO molecules for the hydrogen bond donor groups of the receptor, and the weak basicity of the chloride. This is supported by the stability constant determinations previously reported, and presented in Table 1. Considerable downfield shifts of δ_H7α_ were observed upon addition of acetate, benzoate and bicarbonate anions (Δδ ≈ 2 ppm, Figures 2c–2e), which suggested strong interaction of ureido NH protons with these anions. In addition, the strong deshielding of indolyl H1 protons corroborates its participation in the hydrogen bond formation with carboxylate and bicarbonate moieties (Δδ ≈ 1 ppm). Planar oxo-anions interact both with H1 and H7α due to their Y-shaped geometry. The tetrahedral geometry of the dihydrogen phosphate anion allows strong interaction with all four hydrogen bond donor groups, which is reflected in the substantial deshielding of the H1 and H7α protons (Figure 2f). Interestingly, one set of signals was observed for each type of anion on the NMR timescale, which suggested that the symmetry of the receptor **1** is preserved upon interactions with anions. The stability constant determinations presented in Table 1 also support the finding that this compound interacts selectively with oxo-anions.

Anion–receptor interactions assessed by ^1^H chemical shift changes were corroborated by ^15^N NMR data. Weak shielding of N1 in **1** was observed upon addition of acetate, benzoate and bicarbonate anions, whereas addition of dihydrogen phosphate anions led to deshielding of N1 (Figure 2g). In contrast, N7α was deshielded upon addition of anions (Figure 2g). The strongest deshielding of 11.2 ppm was observed for the **1**·BzO^−^ complex.

#### Conformational properties of **1** and its complexes with anions

**Figure 3:**
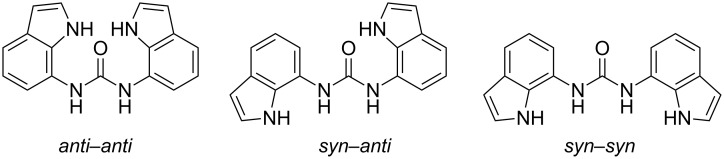
Three representative conformational families of rotamers of **1**. Notations refer to the orientations along [C6–C7–N7α–C7β] fragments.

The rotational flexibility of the ureido moiety allows numerous conformations of receptor **1**. Among them three major, energetically preferred, conformers are likely to be observed (Figure 3). The *syn–syn* conformer, where NH protons form a convergent hydrogen-bonding array, is expected to be adopted in the presence of bound anionic guests, based on the previous solid-state studies. On the other hand, this conformer is unlikely to be abundant in the absence of anions, due to repulsion between the hydrogen bond donor groups. The other two rotamers, namely *anti–anti* and *syn–anti*, can be stabilized by intramolecular NH–CO hydrogen bonds, which represent competition to anion–receptor interactions and therefore make conformational studies even more appealing.

**Figure 4:**
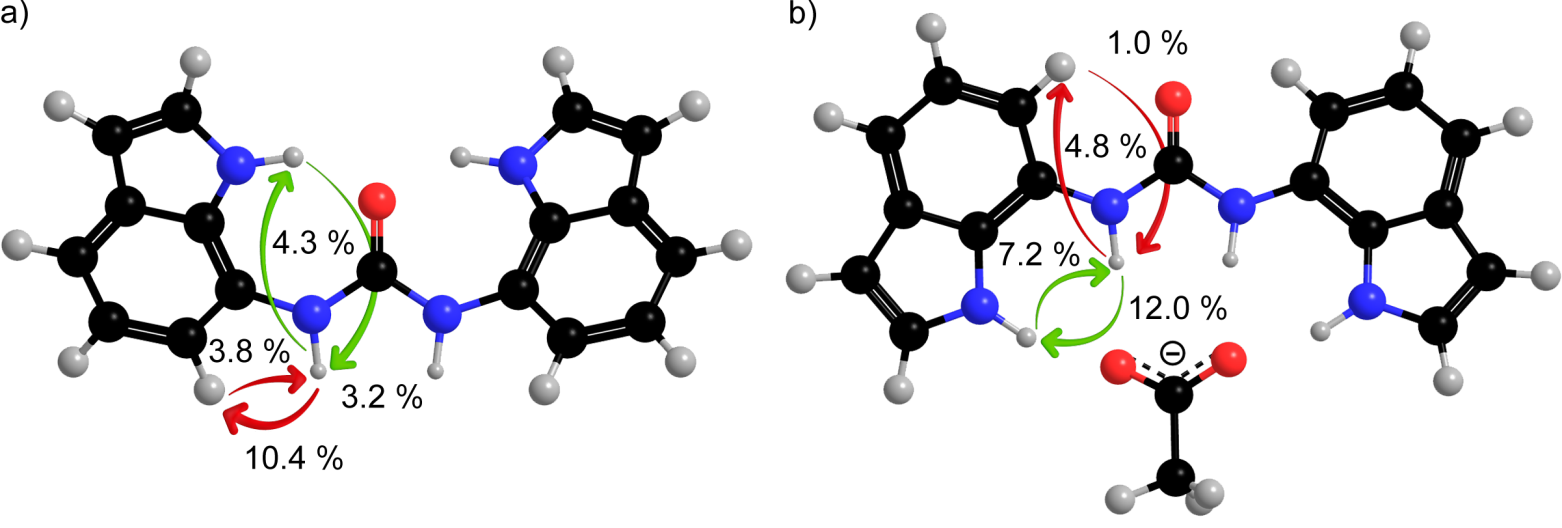
NOE enhancements of **1** in the absence of anions (a) and upon addition of one equivalent of acetate anions (b).

The conformational characteristics of diindolylurea **1** were assessed by the use of 1D difference NOE experiments in the absence and in the presence of anions. The orientation along the C7–N7α bonds was established by comparative evaluation of NOE enhancements of H7α with H1 and H6 protons. The saturation of H7α in **1** gave strong NOE at H6 (10.4%) and moderate NOE at H1 (4.3%), which suggested that the *anti* orientation prevails along the linkage between ureido moiety and indole ring (Figure 4a). As the observed NOE enhancements are primarily a function of the H6–H7α and H1–H7α distances, we compared their values in the optimized *anti*–*anti* (d(H6–H7α) = 2.28 Å) and *syn–syn* (d(H1–H7α) = 2.32 Å) structures and established a minor difference of 0.04 Å which would be reflected in a 1% change in the NOE enhancements. The observed difference between NOE enhancements in the uncomplexed form of receptor **1** was over 6%, which led us to conclude that the *anti*–*anti* conformer is predominant in the DMSO-*d*_6_ solution. In addition, the *anti–anti* conformer of **1** with its plane of symmetry along the carbonyl bond is in agreement with the single set of resonances in the NMR spectra. On the other hand, the *syn*–*anti* rotamer shows a twofold rotational symmetry and is expected to exhibit distinct shielding of nuclei, imposed by the orientations of H6 and H1 protons in the two indole rings with respect to the carbonyl group. However, the populations of the two distinct conformational families are averaged on the NMR time scale.

Only minor changes in the NOE enhancements were observed upon addition of chloride anions to **1**. The strongest NOE was observed between H7α and H6 (9.9%), which was of comparable magnitude to the NOE between the same protons in the absence of anions (Table 3). On the other hand, stronger NOE enhancement between H7α and H1 (7.0%) suggested predominance of the *syn*–*anti* rotamer of **1** in the presence of chloride anions in DMSO-*d*_6_ solution. Interestingly, the *syn*–*syn* rotamer was observed in the crystal structure, where conformational preferences are dictated by other forces, such as crystal packing. These observations are in agreement with minute ^1^H chemical shift changes and the weak stability constant of **1** for the binding of chloride anions.

**Table 3:** Selected NOE enhancements for **1** in the absence and in the presence of different anions (in %).

Saturated:	H1	H6	H7α	

Enhanced:	H7α	H7α	H1	H6

no anion	3.2	3.8	4.3	10.4
Cl^–^	4.2	2.2	7.0	9.9
AcO^–^	7.2	1.0	12.0	4.8
BzO^–^	7.2	0.9	10.4	4.8
HCO_3_^–^	–^a^	0.0	–^a^	4.7
H_2_PO_4_^–^	4.2	0.0	5.3	2.8

^a^Broad NH signals in the baseline.

Considerable changes in the NOE enhancements were observed upon addition of acetate anions to **1**. The saturation of H7α resulted in a much stronger NOE at H1 (12.0%) with respect to H6 (4.8%), which suggested that addition of acetate anions led to conformational changes along the C7–N7α bond (Table 3). The *syn*–*syn* conformer is preferred for the **1**·AcO^−^ complex in solution (Figure 4b). In a similar manner, significant changes in the NOEs were observed upon addition of benzoate anions. The saturation of H7α gave much stronger NOE at H1 (10.4%) with respect to H6 (4.8%, Table 3). Broad NH proton signals prevented the study of the conformation of the **1**·HCO_3_^−^ complex through NOE experiments. NOE enhancements between H1 and H7α (4.2–5.3%) were considerably stronger with respect to NOE between H7α and H6 (0–2.8%) upon addition of dihydrogen phosphate to **1**. The observed NOE enhancements for **1**·H_2_PO_4_^−^ complex suggest a preference for the *syn–syn* conformer in DMSO-*d*_6_.

#### Conformational analysis of **1** and its anion complexes by quantum mechanics calculations

**Figure 5:**
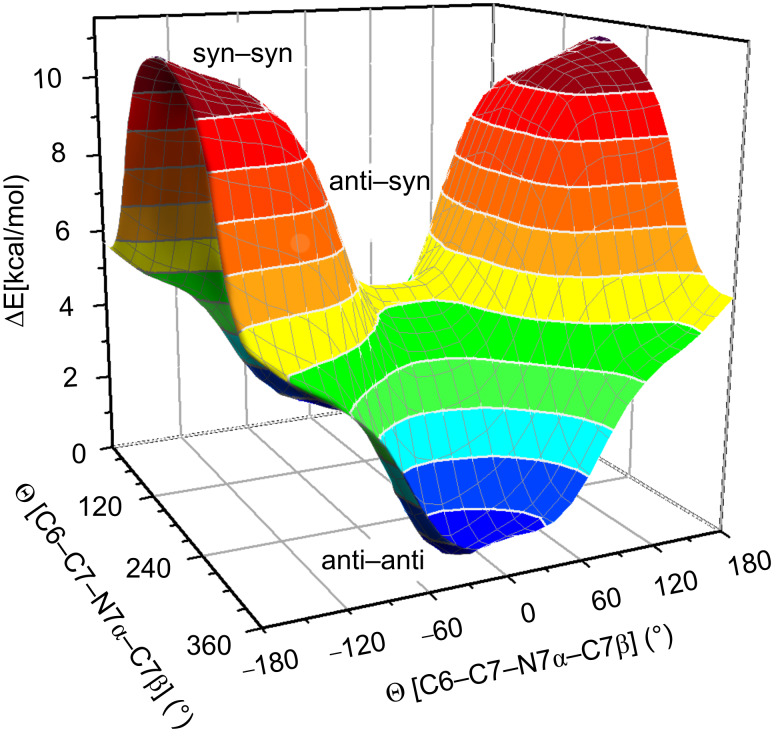
Surface plot of the relative potential energy of **1** as a function of the two constitutive [C6–C7–N7α–C7β] torsion angles. Individual geometries were optimized at the B3LYP/6-311+G(d,p) level of theory at 30° resolution.

The observations on the conformational equilibria in the absence and in the presence of anions were corroborated by quantum mechanical calculations at the B3LYP/6-311+G(d,p) level of theory by means of the Gaussian 03 [40] and Gaussian 09 [41] programs. Indole rings represent the rigid part of the anion receptors, while the substituents on the ureido moiety in **1** exhibit conformational freedom. [C6–C7–N7α–C7β] torsion angles were defined to follow energetic changes induced by reorientation of the indolyl moieties along the C7–N7α bonds. The energy surface for the [C6–C7–N7α–C7β] torsion angles, with 30° resolution, shows that the conformer with the lowest energy is in the *anti*–*anti* region (Figure 5).

**Figure 6:**
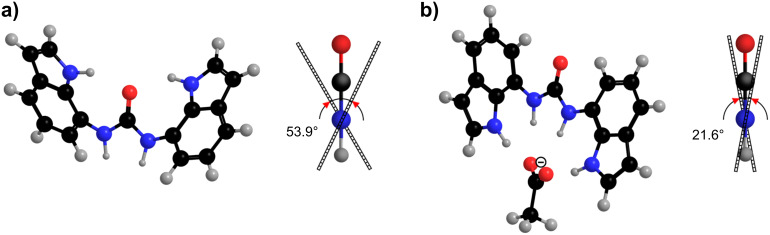
Freely optimized structure at the B3LYP/6-311+G(d,p) level of theory and side view showing deviation from coplanarity defined by the angle between the indolyl rings of receptor **1** in the absence of anions (a) and for the **1**·AcO^−^ complex (b).

Energy minimization of the *anti–anti* conformer of **1** was performed without any constraints, whereas *syn* orientations in the *syn–anti* as well as the *syn–syn* cases were restrained along the [C6–C7–N7α–C7β] torsion angle, while other degrees of freedom were freely optimized. The relative energies for the three representative conformers are reported in Table 4. The *anti–anti* conformer of **1** was found to be the lowest in energy, while the *syn–syn* conformer showed considerably higher energy (11.6 kcal·mol^−1^). The angle between the two indolyl rings in the freely optimized *anti*–*anti* conformer was 53.9° (Figure 6a). The relative energies of the three representative conformers were also computed for complexes of **1** with chloride, acetate and bicarbonate anions. The *syn–syn* conformer exhibited the lowest relative energy for all three anion–receptor complexes (Table 4). The *anti–anti* conformers of anion–receptor complexes exhibited considerably higher energies between 6.2 and 7.3 kcal·mol^−1^. The angle between the two indolyl rings in the freely optimized *syn*–*syn* conformer of the **1**·AcO^−^ complex was found to be 21.6° (Figure 6b). The optimized structure, shown in Figure 6b, shows a single acetate anion bound to the four NH groups in the receptor **1** with N···O distances in the range from 2.75 to 2.95 Å and N–H···O angles in the range 170–176°.

**Table 4:** Relative energies^a^ (in kcal·mol^−1^) of receptor **1** in vacuo and in DMSO, in the absence and in the presence of anions.

anion	conformer	in vacuo	in DMSO

no anion	*anti–anti*	0.00	0.00
	*syn–anti*	5.09	2.74
	*syn–syn*	11.61	6.60

Cl^−^	*anti–anti*	6.50	1.20
	*syn–anti*	1.84	0.12
	*syn–syn*	0.00	0.00

AcO^−^	*anti–anti*	7.26	3.75
	*syn–anti*	2.82	1.74
	*syn–syn*	0.00	0.00

HCO_3_^−^	*anti–anti*	6.21	2.97
	*syn–anti*	2.02	1.31
	*syn–syn*	0.00	0.00


^a^Relative energies are reported with respect to the lowest energy (arbitrarily set to 0.00 kcal/mol) in the absence and in the presence of anions. Geometry optimizations were carried out at B3LYP/6-311+G(d,p).

In order to evaluate the role of DMSO on the energetic preferences of rotamers, relative energies were calculated with the use of Tomasi's polarized continuum model [42–43]. Preferences amongst the three rotamers were retained (Table 4). Only small differences below 1.2 kcal·mol^−1^ were found between the three distinct conformers in the case of the **1**·Cl^−^ complex. In particular, the negligible energy differences between *syn–syn* and *syn–anti* rotamers are in agreement with the NOE data that suggested predominance of the *syn–anti* conformer upon addition of chloride. The energetic preference of the *syn–syn* over the *anti–anti* conformer of 3.8 and 3.0 kcal·mol^−1^ was observed for the **1**·AcO^−^ and the **1**·HCO_3_^−^ complexes, respectively (Table 4). The energetic preferences of the **1**·AcO^–^complex are in excellent agreement with the NOE experiments, which showed conformational reorganizations of **1** upon addition of acetate anions.

#### Conformational features of receptors **2**–**4**

**Figure 7:**
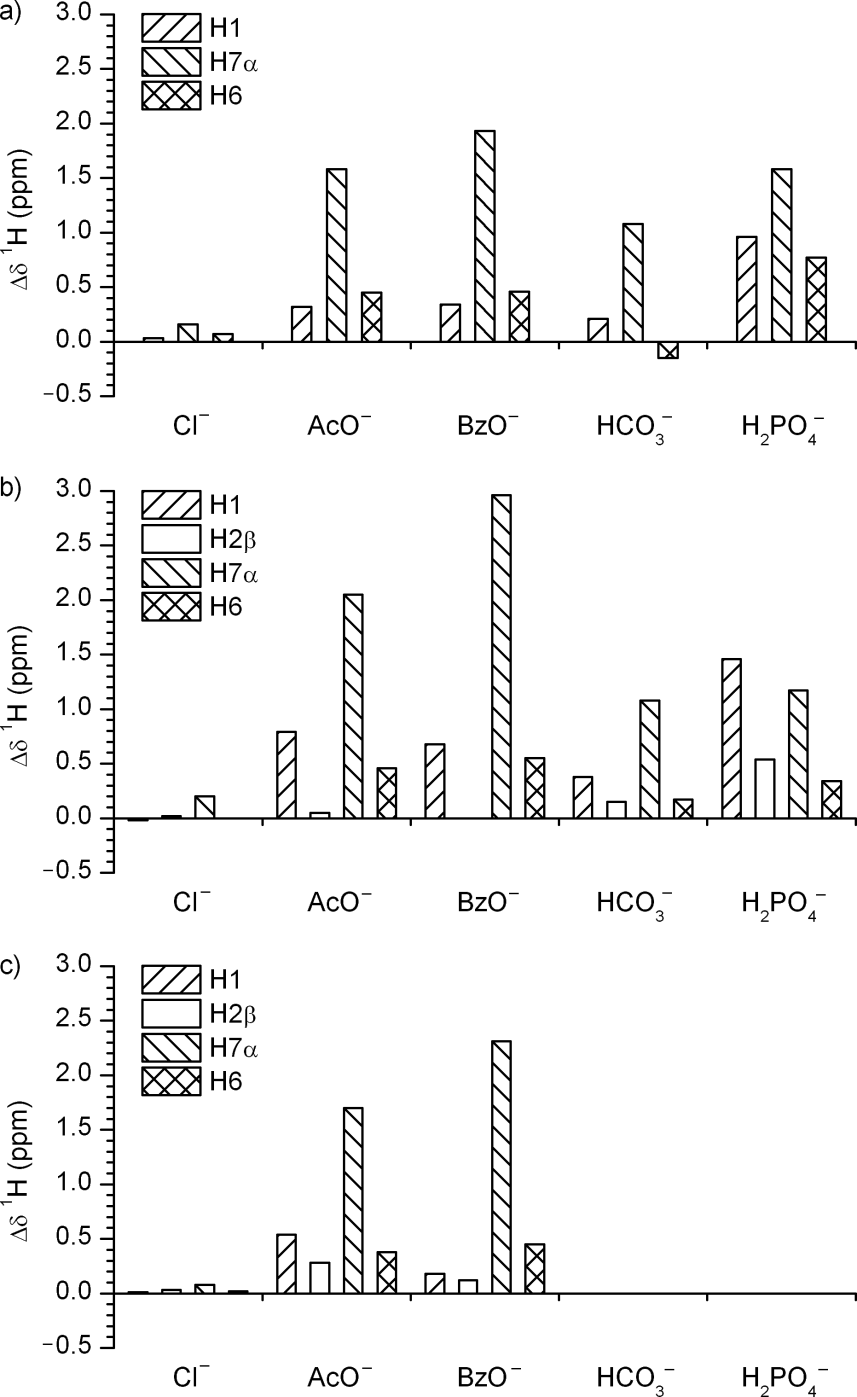
^1^H NMR chemical shift changes, Δδ = δ (in the presence of anions) – δ (in the absence of anions), induced by addition of one equivalent of different anions to receptors **2** (a), **3** (b) and **4** (c). Note, there is no H2β proton in **2**.

**Figure 8:**
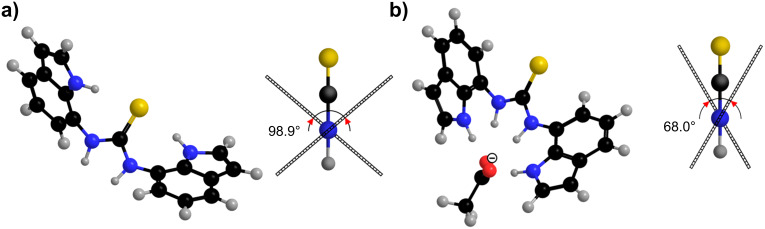
Freely optimized structures at the B3LYP/6-311+G(d,p) level of theory and side view showing deviation from coplanarity defined by the angle between the indolyl rings of **2** (a) and **2**·AcO^−^ complex (b).

The choice of thio (**2** and **4**) versus oxo (**1** and **3**) ureido functionalities, as well as the C2 functionalization of the indole scaffolds with pendant phenyl amides in **3** and **4**, allows tuning of the binding affinities of the receptors. Negligible Δδ values were observed for **2** upon interaction with chloride anions (Figure 7a), which suggests a very weak interaction between chloride and **2**, similar to the weak interactions observed between chloride and **1**. Chemical shift changes showed that the main interaction between receptor **2** and trigonal planar anions (acetate, benzoate and bicarbonate) occurred at the H7α protons (Figure 7a). Addition of dihydrogen phosphate anions caused considerable deshielding of the H1 and H7α protons. Comparison of the Δδ values for **1** and **2** upon interaction with the anions showed that the urea derivative **1** exhibited a higher preference for anion binding relative to thiourea **2** (the data were supported by the stability constant determinations performed previously and shown in Table 1). The larger sulfur atom can prevent the receptor **2** from adopting a planar conformation, which may reduce the affinity of this receptor for anionic guests. Conformational studies of **2** with the use of NOE enhancements showed that the *anti*–*anti* conformer is the preferred conformation in the absence of anions. No apparent conformational changes were observed upon addition of chloride anions to **2**. The overlap of the proton signals as well as the broad line-width of the H1 and H7α NMR resonances prevented conformational analysis of **2** upon addition of other anions used in the study. The conformational preferences of **2** were evaluated by quantum mechanical calculations at the B3LYP/6-311+G(d,p) level of theory. The freely optimized *anti–anti* conformer of **2** exhibited the lowest energy and the *syn–syn* conformer was 8.0 kcal·mol^−1^ higher in energy, in vacuo. Interestingly, the two indolyl rings were not coplanar, with the angle between the two indolyl planes found to be 98.9° (Figure 8a). In the case of **2**·AcO^−^ complex the *syn–syn* conformer exhibited the lowest energy, while the *anti–anti* conformer was 7.4 kcal·mol^−1^ higher in energy, in vacuo. The optimized structure of **2**·AcO^−^ complex is shown in Figure 8b, where the two acetate oxygen atoms are hydrogen bonded to the four NH groups, with N···O distances in the range from 2.76 to 2.94 Å and N–H···O angles in the range 168–177°. The angle between the indolyl rings in the freely optimized *syn*–*syn* conformer of the **2**·AcO^−^ complex was 68.0°.

Bis-amide functionalized diindolylurea **3** exhibits two extra NH groups, which introduces additional possibilities for interactions with anions. The addition of chloride anions to **3** induced negligible chemical shifts, suggesting only weak interactions with this anion (Figure 7b). The strong deshielding of H7α and moderate deshielding of H1 protons in the **3**·AcO^−^ and **3**·BzO^−^ complexes suggests a significant interaction between the anions and the ureido functionality. Interestingly, negligible deshielding of H2β in **3** was observed and this suggests that the amide protons do not participate in the interaction with acetate and benzoate (Figure 7b). The observed Δδ values support the idea that carboxylates were strongly bound to the urea H7α protons which prevented interaction between the anions and the amide H2β protons. Analogously, large chemical shift changes of up to 1 ppm were observed for the H7α and H1 protons upon the addition of bicarbonate anions to **3**. Strong deshielding of H1, H2β and H7α protons in the **3**·H_2_PO_4_^−^ complex suggests that all the NH donor groups are involved in interactions with the dihydrogen phosphate anions (Figure 7b).

**Figure 9:**
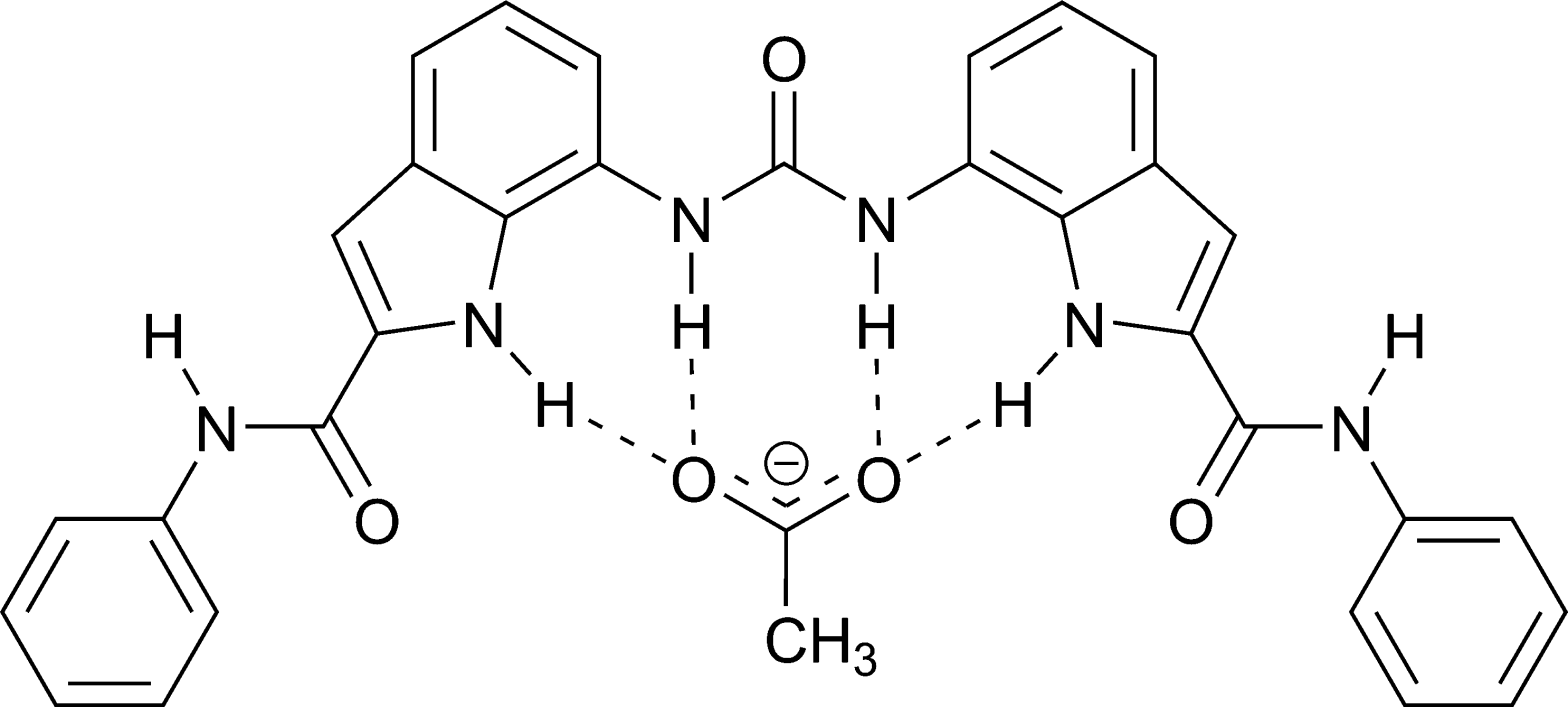
Conformational preferences and proposed binding mode for the **3**·AcO^−^ 1:1 complex.

The conformational properties of **3** and of its complexes with different anions were studied by NOE measurements. The saturation of the H1 protons resulted in moderately negative NOEs at the H7α and H2β protons. The cross peaks in the 2D NOESY spectra between the NH protons and bulk water suggest chemical exchange that complicated the conformational study along the C2–C2α and C7–N7α bonds. Nevertheless, strong NOE enhancements between the H2β and H3 protons suggest an orientation along the C2–C2α bond where the H2β and H3 protons are spatially close and the C2α carbonyl group is oriented towards the indole H1 proton. NOE enhancements between H2β and H3 protons were observed also in the **3**·AcO^−^ and **3**·BzO^−^ complexes, which suggests that the orientation of the carboxamide group along the C2–C2α bond is retained in **3** upon addition of carboxylate anions. This observation was supported by negligible Δδ values for the H2β protons in the **3**·AcO^−^ and **3**·BzO^−^ complexes with respect to **3**. The conformational preferences and the proposed binding mode in the **3**·AcO^−^ complex are shown in Figure 9. A conformational study of **3** in the presence of bicarbonate and dihydrogen phosphate anions was hindered by the broadened and overlapped ^1^H signals. In the solid state compound **3** crystallized with tetrabutylammonium dihydrogen phosphate as the monohydrogen phosphate complex [38].

Only negligible chemical shifts were observed for **4** upon addition of chloride anions (Figure 7c). Considerable deshielding of H7α protons in **4** by up to 2.3 ppm in the **4**·AcO^−^ and **4**·BzO^−^ complexes suggested that the major interactions between carboxylates and receptor **4** occurred at the ureido functionality (Figure 7c). The conformational properties of the **4**·AcO^–^ and **4**·BzO^–^ complexes could not be determined due to the broad and overlapped proton signals. Unfortunately, excessively broad and overlapped ^1^H signals for the **4**·HCO_3_^−^ and **4**·H_2_PO_4_^−^ complexes prevented unambiguous assignment of the NMR resonances and hence the conformational studies of these complexes. In the solid state compound **4** crystallized with tetrabutylammonium dihydrogen phosphate as the monohydrogen phosphate complex (see Supporting Information File 2 for more details).

## Conclusion

The bis-indole receptors **1**–**4** were characterized by heteronuclear NMR spectroscopy. NOE based conformational analysis was supported by quantum mechanics calculations and revealed that diindolylurea **1** exhibited conformational preorganization in DMSO-*d*_6_ solution. The *anti*–*anti* conformer, which could be stabilized by intramolecular hydrogen bonds between the C7β carbonyl group and indole NH proton, was predominant for **1** in the absence of anions. The energetically minimized structure of *anti*–*anti* conformer showed a plane of symmetry along the ureido carbonyl group and deviation from coplanarity amongst the indolyl rings. Anion-induced chemical shift changes suggested weak binding of chloride anions and negligible conformational changes for **1**. Addition of acetate, benzoate, bicarbonate and dihydrogen phosphate resulted in strong deshielding of the ureido protons and moderate deshielding of the indole H1, which indicated that the main hydrogen bond interaction occurred at the urea donor groups of **1**. Furthermore, binding of anions caused conformational changes along the C7–N7α bonds, and the *syn–syn* conformer was predominant in the anion–receptor complexes according to both NOE enhancements and ab initio calculations in solution. The freely optimized *syn*–*syn* conformer of the **1**·AcO^−^ complex retained a plane of symmetry along the carbonyl bond and showed a smaller deviation from indole ring coplanarity than did the *anti*–*anti* conformer of **1**. The conformational preferences for **2** were analogous to those observed for receptor **1**. Unfortunately, excessively broad and overlapped ^1^H signals prevented a detailed conformational analysis of the anion–receptor complexes for **3** and **4**.

## Experimental

### NMR experiments

^1^H, ^13^C and ^15^N NMR spectra were acquired on a Varian Unity Inova 300 MHz NMR spectrometer. All data were recorded in DMSO-*d*_6_ at 298 K. Chemical shifts were referenced to the residual solvent signal of DMSO-*d*_6_ at δ 2.50 ppm for ^1^H (297.801 MHz) and δ 39.50 ppm for ^13^C (76.190 MHz), while ^15^N (30.188 MHz) chemical shifts were referenced relative to external benzamide (δ 103.55 ppm). Individual resonances were assigned on the basis of their chemical shifts, signal intensities, multiplicity of resonances, H–H coupling constants as well as by means of a series of 2D NMR experiments (COSY, gHSQC and gHMBC). The saturation delay in the 1D difference NOE experiment was 5.0 s. All anions were added as tetrabutylammonium salts except bicarbonate which was added as a tetraethylammonium salt. NOESY spectra were acquired with mixing time of 100 and 300 ms.

#### Ab initio calculations

Initial structures were generated by Chem3D Pro 10.0 software and energy minimization at the B3LYP/6-311+G(d,p) level was performed for **1** and **2** without any constraints for the *anti*–*anti* conformers, by means of Gaussian 03 [40] and Gaussian 09 [41]. *Syn* orientations in the *syn–anti* as well as the *syn–syn* conformers of **1** and **2** were restrained along the [C6–C7–N7α–C7β] torsion angle while other degrees of freedom were freely optimized. Ab initio calculations of anion–receptor complexes were carried out without any constraints for the *syn*–*syn* conformers, where anions were placed initially at the expected equilibrium distance to the H1 and H7α protons. The positions of the anions were freely optimized. Energy minimizations of the *syn–anti* and *anti–anti* conformers of the anion–receptor complexes were restrained along the [C6–C7–N7α–C7β] torsion angle while other degrees of freedom were freely optimized. The tetrabutylammonium countercation was omitted in the geometry optimization of the anion–receptor complexes. Frequency calculations verified that the optimized geometries were stable points on the potential energy surface. Relative energies in solution were calculated by means of Tomasi's polarized continuum model, where the dielectric constant of DMSO was used (ε = 46.7).

## Supporting Information

File 1 Experimental for the synthesis of compound **4** and details of the crystal structure of the HPO_4_^2−^ complex of **4**, ^1^H and ^13^C NMR data for **1**–**4,** 1D difference NOE spectra for **1** in the absence and upon addition of one equivalent of acetate anions.

File 2 Crystallographic data of the complex of compound **4** with tetrabutylammonium dihydrogen phosphate (**4**·TBA_2_·HPO_4_).
