# Supplementary material for: NMR studies of anion-induced conformational changes in diindolylureas and diindolylthioureas
Source: Beilstein J Org Chem. 2011 Sep 2;7:1205–14. doi: 10.3762/bjoc.7.140 (PMC3182429; doi:10.3762/bjoc.7.140)
Supplement: File 1 — Experimental for the synthesis of compound 4 and details of the crystal structure of the HPO42− complex of 4, 1H and 13C NMR data for 1–4, 1D difference NOE spectra for 1 in the absence and upon addition of one equivalent of acetate anions. [file Beilstein_J_Org_Chem-07-1205-s001.pdf]

Supporting Information File 1

for

NMR studies of anion-induced conformational changes in diindolylureas and diindolylthioureas

Damjan Makuc[1,2], Jennifer R. Hiscock[3], Mark E. Light[3], Philip A. Gale*[3] and Janez Plavec*[1,2,4]

Address: [1]Slovenian NMR Centre, National Institute of Chemistry, Hajdrihova 19, SI-1000 Ljubljana, Slovenia, [2]EN→FIST Centre of Excellence, Dunajska 156, SI-1000 Ljubljana, Slovenia, [3]Chemistry, University of Southampton, Southampton SO17 1BJ, United Kingdom and [4]Faculty of Chemistry and Chemical Technology, University of Ljubljana, SI-1000 Ljubljana, Slovenia

Email: Damjan Makuc - damjan.makuc@ki.si; Jennifer R. Hiscock - J.Hiscock@soton.ac.uk; Mark E. Light - m.e.light@soton.ac.uk; Philip A. Gale* - philip.gale@soton.ac.uk; Janez Plavec* - janez.plavec@ki.si

* Corresponding author

Experimental for the synthesis of compound 4 and details of the crystal structure of the HPO_4^{2-} complex of 4, ^{1}H and ^{13}C NMR data for 1–4, 1D difference NOE spectra for 1 in the absence and upon addition of one equivalent of acetate anions.

General remarks: All reactions were performed in oven dried glassware under slight positive pressure of nitrogen/argon (as specified). ^{1}H NMR (300 MHz) and ^{13}C{1H} NMR (75 MHz) spectra were determined on a Bruker AV300 spectrometer. ^{1}H NMR (400 MHz) and ^{13}C NMR (100 MHz) spectra were determined on a Bruker AV400 spectrometer. Chemical shifts for 1H NMR are reported in parts per million (ppm), calibrated to the solvent peak set relative to the singlet at δ = 2.5 ppm for deuterio-dimethylsulfoxide, with coupling constants reported in Hertz (Hz). The following abbreviations are used for spin multiplicity: s = singlet, d = doublet, t = triplet, m = multiplet. Chemical shifts for ^{13}C{^{1}H} NMR are reported in ppm, relative to the central line of a septet at δ = 39.52 ppm for deuteriodimethylsulfoxide. Infrared (IR) spectra were recorded on a Matterson Satellite (ATR). FTIR are reported in wavenumbers (cm^{-1}). All solvents and starting materials were purchased from chemical sources where available.

7,7'-(Thiocarbonylbis(azanediyl))bis(*N*-phenyl-1*H*-indole-2-carboxamide) (4)

7-Nitro-*N*-phenyl-1*H*-indole-2-carboxamide was synthesized by a previously reported method [1]. 7-Nitro-*N*-phenyl-1*H*-indole-2-carboxamide (0.30 g, 1.07 mM) and a Pd/C 10% catalyst (0.02 g) were suspended in ethanol (25 mL). The flask was evacuated and the mixture placed under a hydrogen atmosphere and stirred vigorously for 6 h. After this time the palladium catalyst was removed by filtration through celite and the filtrate taken to dryness and placed under reduced pressure. This gave a white solid. Assumed yield 100%.

7-Amino-*N*-phenyl-1*H*-indole-2-carboxamide (0.27 g, 1.07 mM) was dissolved in a mixture of chloroform (20 mL) and a saturated aqueous solution of $NaHCO_3$ (25 mL). Thiophosgene (0.05 mL, 1.07 mM) was added dropwise in chloroform (5 mL) and the reaction mixture was left stirring overnight under a nitrogen atmosphere at room temperature. The organic layer was washed with water, dried over $MgSO_4$, filtered and concentrated in vacuo. The pure product was obtained by recrystallization from hot MeOH. The isothiocyanate was isolated as a white solid and taken straight on to the next reaction: Assumed yield 100%.

A solution of 7-amino-*N*-phenyl-1*H*-indole-2-carboxamide (0.27 g, 1.07 mM) and 7-isothiocyanato-*N*-phenyl-1*H*-indole-2-carboxamide (0.31 g, 1.07 mM) in pyridine

(20 mL) was stirred overnight under nitrogen. The pyridine was then removed and the oil dissolved in hexane (5 mL). The solid was collected by filtration, sonicated in ethylacetate (50 mL) for 30 min and then heated at reflux for 5 h. The solution was then reduced in volume to 10 mL and allowed to cool. A white solid was collected by filtration and dried under vacuum. Yield 27%; mp. 197 °C; ^{1}H NMR (300 MHz, DMSO-d_6): δ: 7.07–7.13 (m, 2H), 7.34–7.41 (m, 3H), 7.48 (d, J = 1.83 Hz, 1H), 7.57 (d, J = 8.04 Hz, 1H), 7.84 (d, J = 7.68 Hz, 2H), 9.73 (s, NH, 1H), 10.27 (s, NH, 1H), 11.68 (s, NH, 1H); ^{13}C NMR (75 MHz, DMSO-d_6): δ: 105.1 (ArCH), 119.5 (ArCH), 120.1 (ArCH), 120.9 (ArCH), 123.6 (ArCH), 124.6 (ArC), 128.7 (2 × ArCH), 131.7 (ArC), 132.2 (ArC), 138.9 (ArC), 159.5 (CO), 180.8 (CS); IR (film): ν = 3320 (indole NH stretching), 3300 (thiourea NH stretching), 1630 and 1530 (amide CO) stretching), 1250 (thiourea CS stretching); LRMS (ES$^+$) m/z: 567 [M + Na]$^+$;
HRMS (ES$^+$) m/z: act: 567.1586 [M + Na]$^+$cal: 567.1574 [M + Na]$^+$

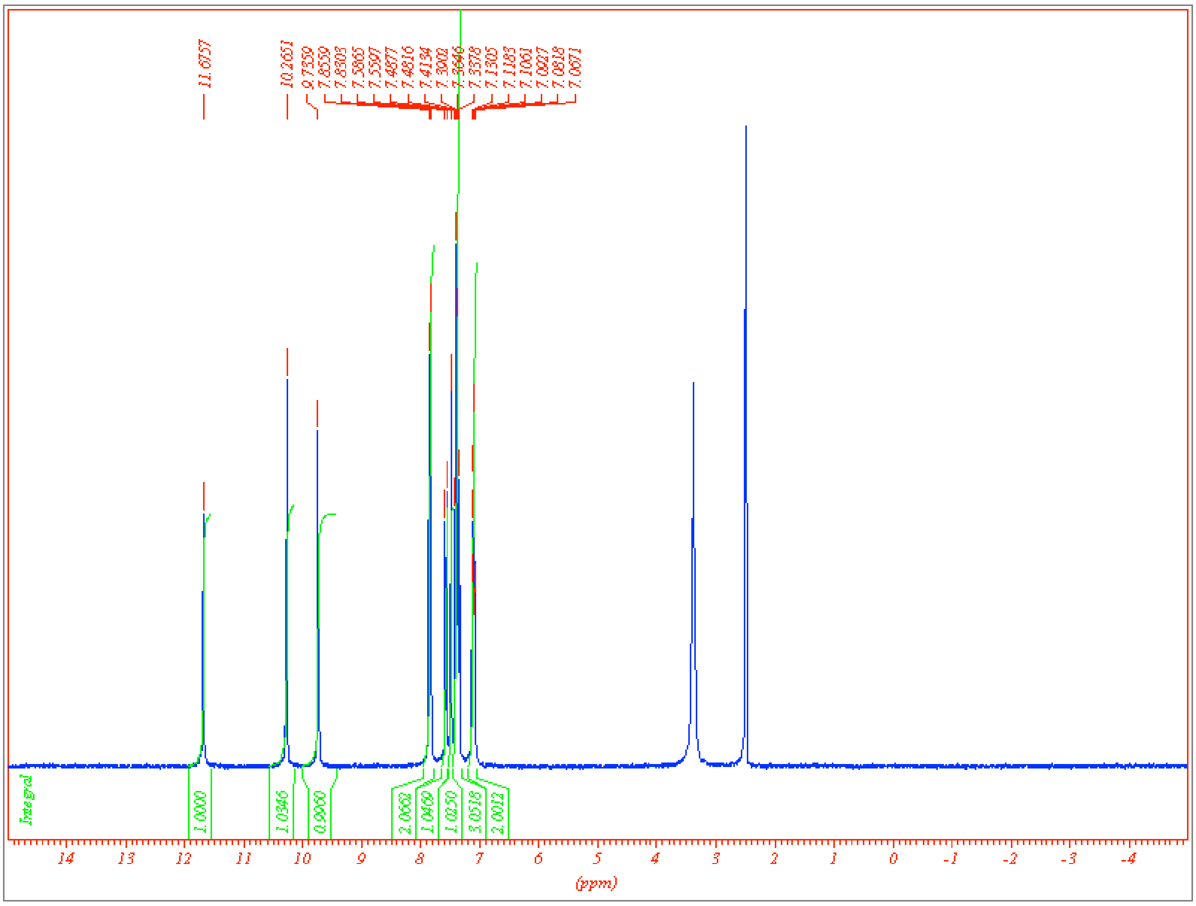

Figure S1: ^{1}H NMR spectrum of compound **4** in DMSO-d_6.

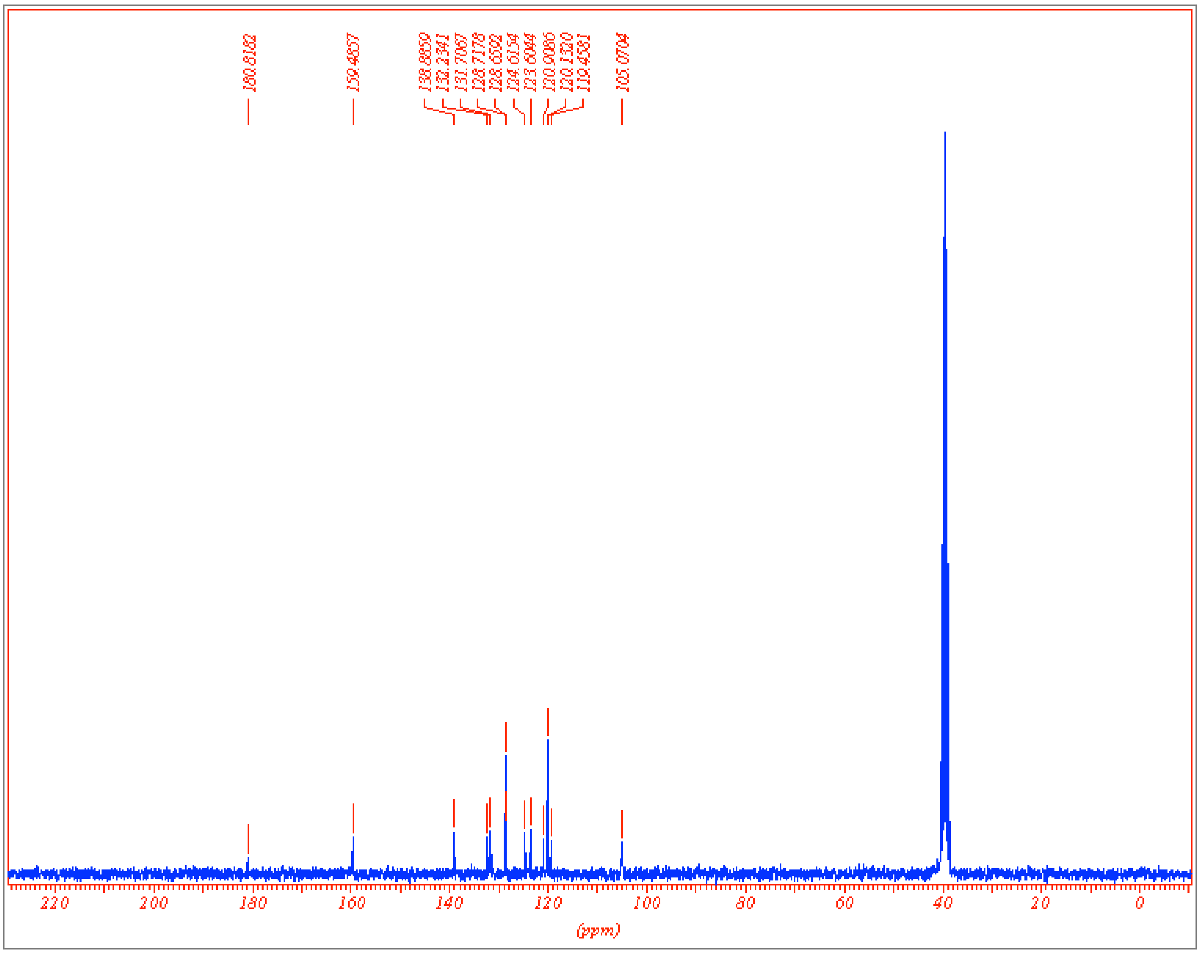

Figure S2: ^{13}C NMR spectrum of compound **4** in DMSO-d_6. One signal is missing due to the overlapping of signals.

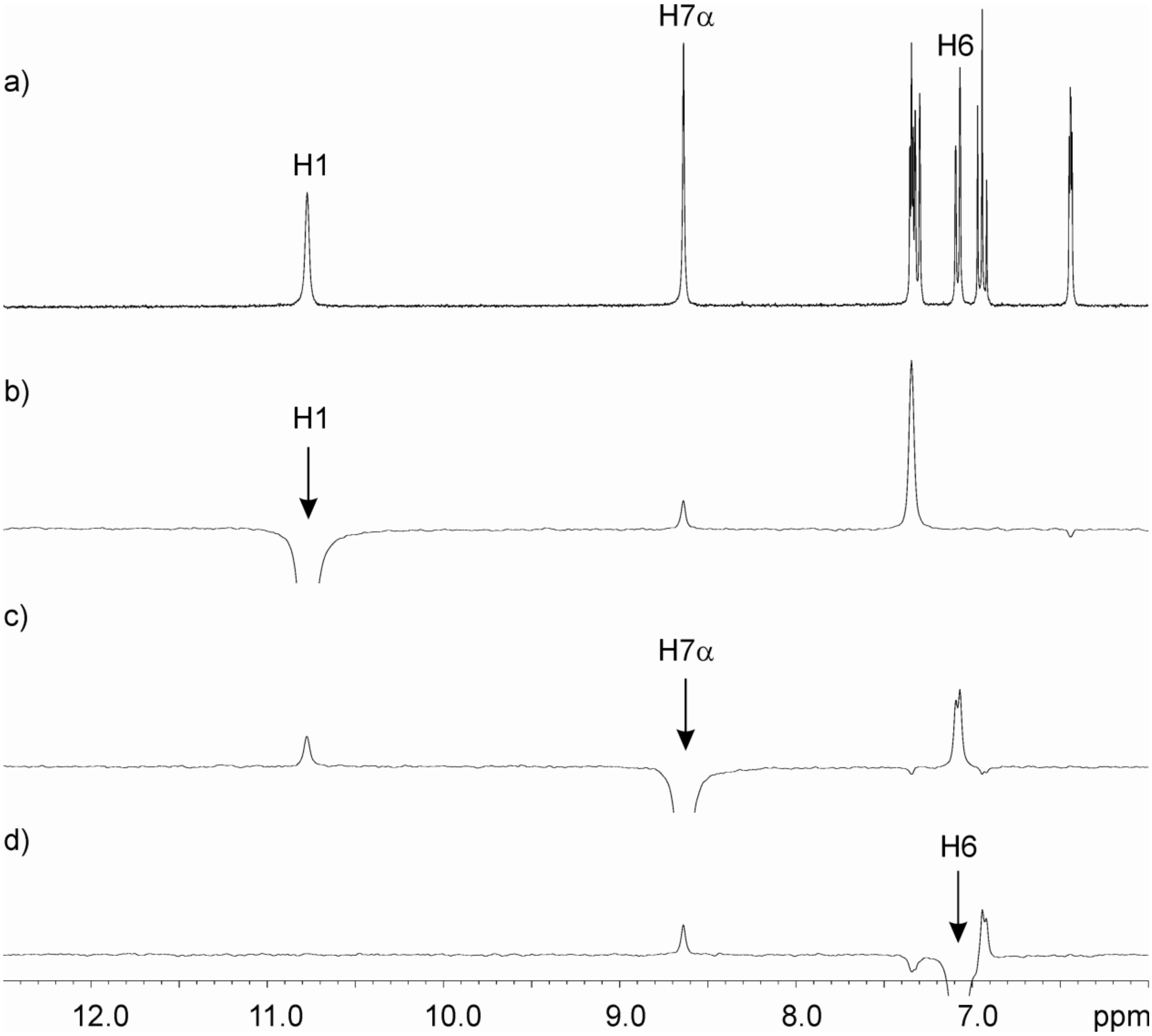

Figure S3: ^{1}H NMR spectra of **1** in the absence of anions (a) and corresponding 1D difference NOE spectra upon saturation of H1 (b), H7α (c) and H6 protons (d). All spectra were acquired in acetone-d_6 at 298 K.

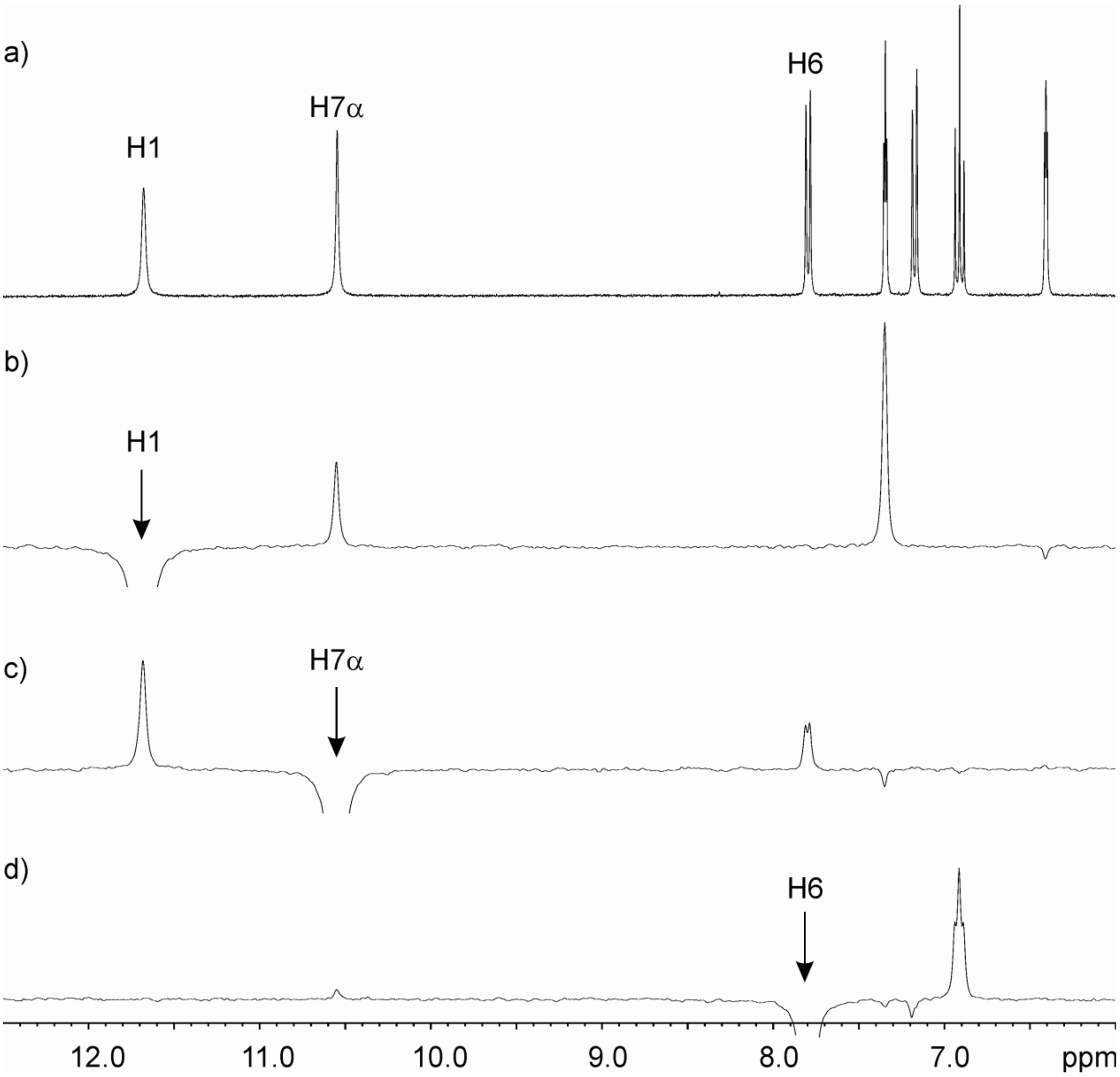

Figure S4: ^{1}H NMR spectra of **1** upon addition of one equivalent of acetate anions (a) and corresponding 1D difference NOE spectra upon saturation of H1 (b), H7α (c) and H6 protons (d). All spectra were acquired in acetone-d_6 at 298 K.

X-ray data

X-ray data were collected on a Bruker Nonius APEXII detector mounted at the window of a Mo rotating anode generator – standard procedures were followed. Crystal data for the monohydrogen phosphate complex of compound **4**: M = 1125.52, Triclinic, a = 11.3537(3), b = 15.5301(4), c = 18.6701(5) Å, α = 81.989(2)°, β = 76.082(2)°, γ = 78.3380(10)°, U = 3115.09(14) Å^3, T = 120(2) K, space group $P-1$, Z = 2, μ = 0.133 mm^{-1}, 70303 reflections measured, 14304 unique reflections (R_{int} = 0.1004). The final R_1 values were 0.0781 ($I > 2\sigma(I)$). The final $wR(F_2)$ values were 0.1777 ($I > 2\sigma(I)$). The final R_1 values were 0.1428 (all data). The final $wR(F_2)$ values were 0.2071 (all data). The goodness of fit on F_2 was 1.032.

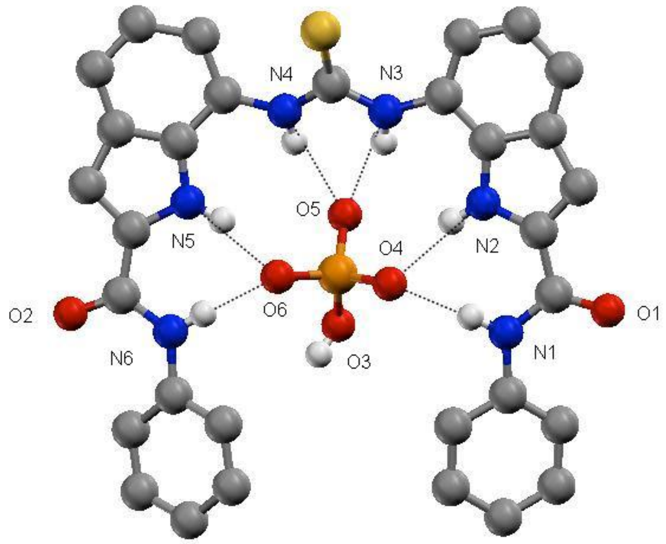

Figure S5: Single crystal X-ray structure of compound **4** with TBA_2 HPO_4. The TBA counter cations and aromatic hydrogen atoms have been omitted for clarity. The crystal structure was obtained by slow evaporation of a solution of compound **4**, TBA H_2PO_4 in DMSO. X-ray data were collected on a Bruker Nonius APEXII detector mounted at the window of a Mo rotating anode generator – standard procedures were followed. Crystal data for compound **4-**TBA_2 HPO_4: M = 1125.52, Triclinic, a = 11.3537(3), b = 15.5301(4), c = 18.6701(5) Å, α = 81.989(2)°, β = 76.082(2)°, γ = 78.3380(10)°, U = 3115.09(14) Å^3, T = 120(2) K, space group P−1, Z = 2, μ = 0.133 mm^{-1}, 70303 reflections measured, 14304 unique reflections (R_{int} = 0.1004). The final R_1 values were 0.0781 ($I > 2\sigma(I)$). The final $wR(F_2)$ values were 0.1777 ($I > 2\sigma(I)$). The final R_1 values were 0.1428 (all data). The final $wR(F_2)$ values were 0.2071 (all data). The goodness of fit on F_2 was 1.032.

^{1}H and ^{13}C NMR data for 1–4

1,3-Bis(1*H*-indol-7-yl)urea (1)

δH (300 MHz; DMSO-d_6) 6.44 (H3), 6.94 (H5), 7.08 (H6), 7.31 (H4), 7.35 (H2), 8.64 (H7α), 10.78 ppm (H1); *δ*C (75 MHz; DMSO-d_6) 101.5 (C3), 113.7 (C6), 115.9 (C4), 119.0 (C5), 124.0 (C7), 125.1 (C2), 129.0 (C7a), 129.4 (C3a), 153.6 ppm (C7β).

1,3-Bis(1*H*-indol-7-yl)thiourea (2)

δH (300 MHz; DMSO-d_6) 6.46 (H3), 6.96 (H5), 7.03 (H6), 7.36 (H2), 7.44 (H4), 9.48 (H7α), 11.03 ppm (H1); *δ*C (75 MHz; DMSO-d_6) 101.5 (C3), 118.4 (C4), 118.8 (C5), 119.2 (C6), 123.7 (C7), 125.3 (C2), 129.3 (C3a), 132.0 (C7a), 180.7 ppm (C7β).

7,7'-(Carbonylbis(azanediyl))bis(*N*-phenyl-1*H*-indole-2-carboxamide) (3)

δH (300 MHz; DMSO-d_6) 7.07 (H5), 7.12 (H2ζ), 7.38 (H2ε), 7.40 (H4), 7.49 (H3), 7.59 (H6), 7.82 (H2δ), 8.97 (H7α), 10.29 (H2β), 11.62 ppm (H1); *δ*C (75 MHz; DMSO-d_6) 104.5 (C3), 114.3 (C6), 116.3 (C4), 120.2 (C2δ), 120.5 (C5), 123.6 (C2ζ), 125.0 (C7), 128.5 (C3a and C7a), 128.7 (C2ε), 131.2 (C2), 138.8 (C2γ), 153.1 (C7β), 159.6 ppm (C2α).

7,7'-(Thiocarbonylbis(azanediyl))bis(*N*-phenyl-1*H*-indole-2-carboxamide) (4)

δH (300 MHz; DMSO-d_6) 7.09 (H5), 7.11 (H2ζ), 7.36 (H2ε), 7.39 (H6), 7.48 (H3), 7.57 (H4), 7.84 (H2δ), 9.72 (H7α), 10.26 (H2β), 11.68 ppm (H1); *δ*C (75 MHz; DMSO-d_6) 105.1 (C3), 119.5 (C4), 120.2 (C5 and C2δ), 121.0 (C6), 123.7 (C2ζ), 124.6 (C7), 128.7 (C3a and C2ε), 131.7 (C2), 132.3 (C7a), 138.9 (C2γ), 159.5 (C2α), 180.8 ppm (C7β).

Reference

1. Bates, G. W.; Triyanti; Light, M. E.; Albrecht, M.; Gale, P. A. *J. Org. Chem.* **2007,** *72,* 8921–8927. doi:10.1021/jo701702p
